# Effect of Health Literacy on Antiviral Treatment of Hepatitis B: Instrumental Variable Analysis

**DOI:** 10.2196/58391

**Published:** 2024-12-16

**Authors:** Hanchao Cheng, Shiyang Liu, Zhitao Wang, Qiyou Wu, Xin Wang, Polin Chan, Zhongdan Chen, Simon Luo, Yinghua Li, Jing Sun

**Affiliations:** 1 School of Health Policy and Management, Chinese Academy of Medical Sciences and Peking Union Medical College, Beijing, China; 2 Medical School of Chinese People's Liberation Army, The Fifth Medical Center of People's Liberation Army General Hospital, Beijing, China; 3 Tongji Hospital, Tongji Medical College, Huazhong University of Science & Technology, Wuhan, China; 4 Hepatitis/Tuberculosis/HIV/STI, World Health Organization Regional Office for the South East Asia, Dehli, India; 5 Hepatitis/TB/HVI/STI, World Health Organization Representative Office in China, Beijing, China; 6 IQVIA, China, Beijing, China; 7 China Health Education Center, Beijing, China

**Keywords:** health literacy, antiviral treatment, hepatitis B, China, instrumental variable, longitudinal study

## Abstract

**Background:**

China is a country with a high burden of hepatitis B (Hep B) but a low treatment rate. One of the key reasons for the low treatment rate is the inadequate health literacy (HL) of the people, which may affect the awareness and knowledge of Hep B and its treatment, as well as the ability to actively and correctly seek medical resources.

**Objective:**

This study analyzed how HL contributed to the scale-up of antiviral treatment of Hep B in China. We expect that the findings of this study could be used to inform resource allocation for health education and other approaches intending to improve the HL of the Chinese population, thus facilitating the nationwide scale-up of Hep B treatment and contributing to the achievement of the 2030 goal of eliminating viral hepatitis as a major public health threat in China.

**Methods:**

We used the two-stage least squares regression method and adopted the mobile phone penetration rate as the instrumental variable to estimate the effect of improved HL on the number of 12-month standard Hep B antiviral treatments in China based on the panel data of 31 provinces from 2013 to 2020.

**Results:**

In the cross-sectional dimension, the higher the HL, the higher the number of treatments in the provinces in a specific year. In the time series dimension, the number of treatments in a specific province increased with the improvement of HL over time. After controlling the time-invariant inherent attributes of provinces, the instrumental variable estimation with two-stage least squares regression based on the province fixed effect model found that for every 1% increase of HL in each province, the number of treatments increased by 7.15% (0.0715 = e^0.0691^ – 1; *P*<.001). Such an increase turned to 5.19% (0.0519 = e^0.0506^ – 1; *P*<.001) for the analysis targeting the observation time from 2013 to 2019, as the data of 2020 were removed when the COVID-19 pandemic started. The study found no statistically significant effect of HL on the number of Hep B treatments in the provinces with higher newly reported Hep B incidence and lower gross domestic product per capita.

**Conclusions:**

Our findings suggest that improved HL of the population is an important favorable facilitator for the scale-up of Hep B treatment in China. Building awareness and knowledge of Hep B and its treatment can help individuals understand their health status, ensuring a healthier lifestyle and appropriate health care–seeking behaviors and health care service utilization, so that people can be diagnosed and treated timely and appropriately. Enhancing resource allocation to improve the overall HL of the population and sending Hep B–specific messages to the infected people would be a feasible and effective approach to scale-up the treatment of Hep B in low- and middle-income settings with limited resources, and contribute to achieving the 2030 global goal of eliminating viral hepatitis as a major public health threat.

## Introduction

China is a country with a high burden of hepatitis B (Hep B), with an estimated 75 million people living with Hep B, accounting for one-third of the global cases [1]. There will be about 10 million people dying from Hep B–related diseases by 2030 if no additional measures are taken [2]. To achieve the global goal of eliminating viral hepatitis as a major public health threat by 2030 set by the World Health Organization, 90% of people infected with hepatitis B virus (HBV) need to be diagnosed and 80% of the eligible patients need to be treated [3]. However, only 19% (16,085,000/86,007,000) of people infected with HBV have been diagnosed and 11% (3,500,000/32,315,000) have been treated in China, which is far below the 2030 targets [1].

**Figure 1.**
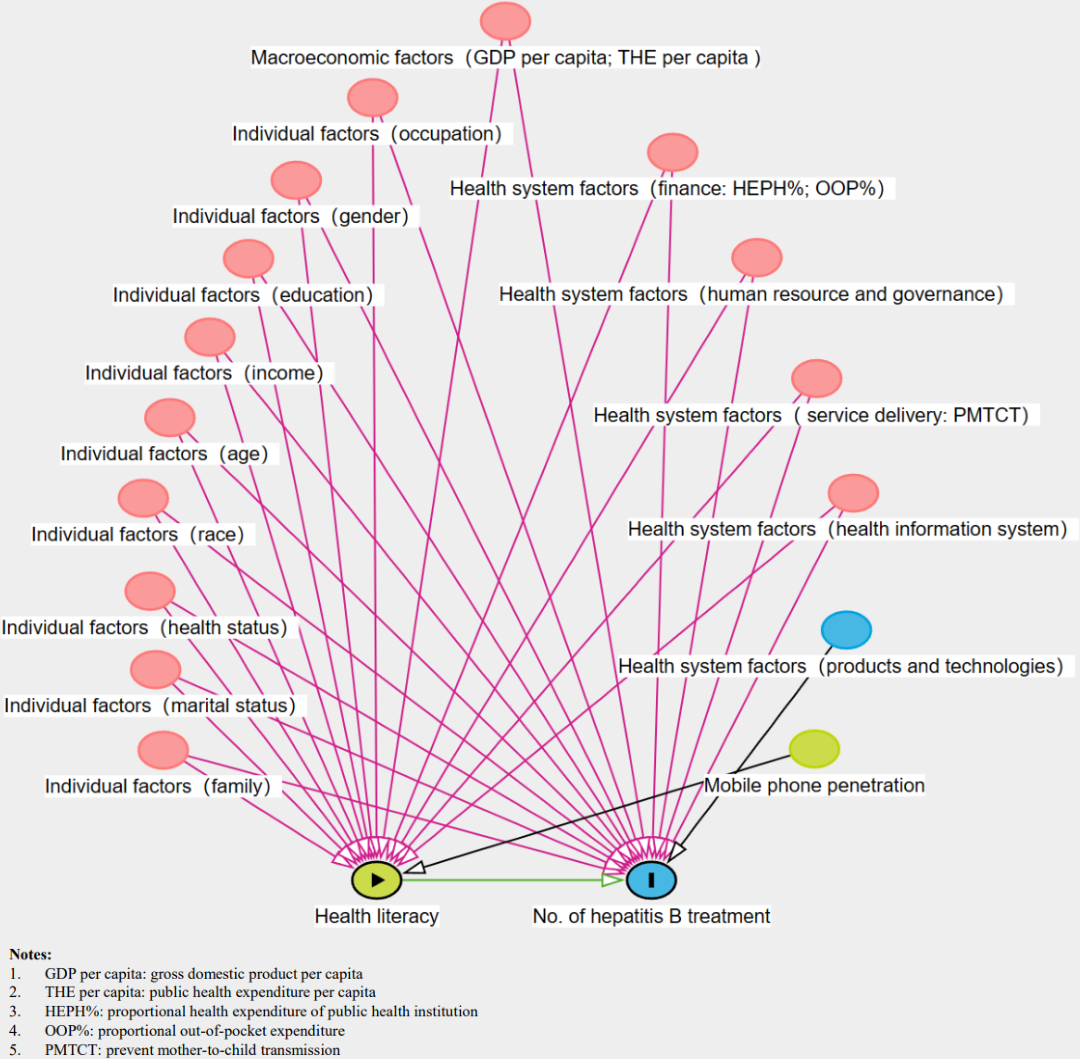
Factors associated with the number of hepatitis B treatments and health literacy.

Health literacy (HL), as defined by the Chinese Health Literacy Scale, has several key components such as the awareness and knowledge of health, the healthy lifestyle and behaviors, as well as the ability to actively and correctly seek medical resources, and utilize health care services aiming to maintain and promote health [4]. A low level of HL could have implications for treatment and was associated with adverse disease outcomes, mainly due to its negative influence on treatment compliance and quality of care [5-9]. Thus, we assumed that HL is a crucial factor for the scaling-up of Hep B treatments. There are three levels of factors that influence HL and Hep B treatment, including individual factors, health system factors, and macroenvironment factors [10-16], as shown in Figure 1. HL interventions always occur within communities or worksites, and interventions targeting the population could be cost-effective in promoting Hep B treatment [17]. There has been a long tradition of HL research in high-income countries but a lack of research in low- and middle-income countries [18-20]. No published studies in China assessed how the HL of individuals affects Hep B treatment.

Based on the panel data of 31 provinces of China from 2013 to 2020, this study aimed to validate the potential effect of improved HL of the Chinese population at the provincial level on the scale-up of Hep B treatment. We expect that the development of this evidence could provide a new perspective for the prevention and treatment of chronic diseases, affirming the necessity of residents’ health education and improving their HL. We hope that the findings of this study can be used to inform resource allocation for health education and other approaches intending to improve the HL of the Chinese population, thus facilitating the nationwide scale-up of Hep B treatment and contributing to the achievement of the 2030 goal of the elimination of viral hepatitis as a major public health threat in China.

## Methods

### Ethical Considerations

All analyses of this study were based on the existing data, and all data were not associated with individuals but aggregated for the provinces. According to Article 32 of the Measures for the Ethical Review of Life Science and Medical Research Involving Humans-2023, where the use of human information and data to carry out life science or medical research involving human beings does not cause harm to the human body, does not involve sensitive personal information, or commercial interests, ethics review may be waived. Therefore, ethics approval was not required [21].

### Data Source

We extracted the annual consumption volume of all six nucleoside/nucleotide analogs (NAs) approved for chronic Hep B antiviral treatment in China and the supplier information of each NA in each province during 2013 and 2020 from IQVIA’s China Hospital Pharmacy Audit (CHPA) database (Table S1 in Multimedia Appendix 1). These six NAs are recommended by the national guidelines for the treatment of Hep B and are covered by the national health insurance. Their specific dosages and strengths correspond to a single indication of Hep B. Only tenofovir has a single strength and dosage form but two indications, Hep B and AIDS. Considering that China implements the naitonal policy for free antiviral treatment AIDS, the antivirals for AIDS treatment are procured and managed by the Center for Disease Control and Prevention through a separate procurement channel. This enables us to measure the number of standard antiviral treatments for Hep B based on the consumption of these six NAs. The CHPA dataset contains longitudinal consumption data of the volume and value of all marketed medicines collected from hospitals with more than 100 beds across 31 provinces in China.

HL has been evaluated by the China Health Education Center using the Chinese Health Literacy Scale in 31 provinces through the annual household survey since 2012 [22]. We obtained the overall HL of the people in each province from 2013 to 2020 from the official websites of provincial governments and health authorities, as well as published literature. We also contacted relevant professional institutions for supplementary data. Provincial mobile phone penetration was extracted from the National Bureau of Statistics [23]. Data on other variables were obtained from the China Health Statistics Yearbook [24] and the China Health Expenditure Research Report [25].

### Study Design

The study design was inspired by the positive relationship between the improved HL and an increased number of Hep B treatments across 31 provinces (Figure S1 in Multimedia Appendix 1). As it is difficult to acquire and measure the comprehensive influencing factors of HL and Hep B treatment, and because HL and Hep B treatment are correlated with each other, the verification of the potential effect must address the issue of endogeneity. We adopted the instrumental variable (IV) method and the two-stage least squares (2SLS) regression, which intended to divide the treatment variable into two parts, the part that is associated and the part that is not associated with the error term. The potential effect could be estimated by using the part that is not associated with the error term [26]. A valid IV must hold the following assumptions: the IV is correlated with the treatment variable, the IV is not correlated with the error term, and the IV can only affect the outcome variable by affecting the treatment variable (exclusion restriction) [27,28]. Health education is one of the main approaches to improve HL, but it is far from adequate and efficient in Chinese settings. Traditional media such as newspapers, radio, and television have been increasingly substituted by new media sources. The mobile phone has become the most used channel for Chinese citizens to access health information [29]. Increased mobile phone penetration may help to improve HL through efficient health information dissemination, health education, and patient management, thus facilitating the scale-up of Hep B treatment. More importantly, mobile phone penetration does not affect Hep B treatment directly but plays an indirect role in improving HL. Although increased mobile phone penetration might be associated with the economic and technological developments, which might promote the advancement of diagnostics and treatment of Hep B. Considering that there are multiple cost-effective diagnostics and treatments available in China, new options might not play a critical role in increasing the number of Hep B treatment. We took provincial mobile phone penetration as an IV to estimate the potential effect of HL on the number of Hep B treatments based on the national panel data from 2013 to 2020. Detailed explanations of the choice of the IV are presented in Supplementary Material S1 in Multimedia Appendix 1.

### Measurement

As the number of patients with Hep B eligible for treatment is high, the data are not available at the provincial level, and the treatment rate is very low, we adopted the number of Hep treatments rather than the treatment rate as the key outcome variable. We transformed the annual consumption volume of each NA recommended by the Chinese guidelines for Hep B antiviral treatment into the number of 12-month standard treatments, by idealizing and simplifying the complicated situation of treatment in the real world. The transformation followed the treatment course and dosage defined by the national guidelines and official pharmaceutical manufacturers’ instructions. In addition, we adopted the annual number of suppliers of NAs for Hep B treatment in each province from 2013 to 2020 as the outcome variable for the falsification test.

HL was evaluated using the Chinese Health Literacy Scale, with a total score of 100. If the evaluation score was ≥80, the survey respondent was judged to be having appropriate level of HL. The level of HL in each province was presented as the proportionate number of residents with the appropriate level of HL. Each province adopted the stratified multistage probability proportionate to size sampling (PPS) method to target the residents aged between 15 and 69 years [4].

Mobile phone penetration was measured as the number of mobile phones per 100 people. We used the total population and the reported prevalence of Hep B in each province as covariates for all the models, which would be able to help control the number of infected people eligible for antiviral treatment in each province to a certain extent. The gross domestic product (GDP) per capita and the total health expenditure (THE) per capita were the covariates to control the socioeconomic environment. The proportionate health expenditures of public health institutions and the proportionate out-of-pocket (OOP%) were the covariates to control the health financing status from the perspectives of public inputs in public health institutions (including health education and health promotion services) and individual financial burden of health care services.

### Statistical Analysis

We used the pooled ordinary least square (OLS) regression method to roughly estimate the effect of HL on the number of 12-month standard treatments of Hep B as a baseline reference with model 1. We then adopted the province fixed effect model to control the unobserved time-invariant inherent attributes among provinces (model 2), and the year fixed effect model to control the time-varying factors (model 3) [30]. The IV estimation with 2SLS based on the province fixed effect model was performed to minimize the endogeneity. In the first stage of the regression, HL was the outcome variable and IV (mobile phone penetration) was the treatment variable; thus, we obtained the fitting value of HL (model 4‐1). In the second stage of regression, the number of treatments was the outcome variable and the fitting value of HL we got from the first stage was the treatment variable (model 4‐2). Detailed explanations of the models are presented in Supplementary Material S1 in Multimedia Appendix 1.

We performed the least square dummy variable to test whether there were differences among provinces by adding dummy variables of 31 provinces (Table S2 in Multimedia Appendix 1). The *F* test was applied to check whether there was a time effect. We did not adopt a 2-way fixed effects model considering that there is no practical meaning in this study.

We conducted the following tests to verify the rationality of the IV method: (1) the Durbin-Wu-Hausman endogeneity test to check if there are endogenous variables, and the necessity to adopt the IV method; (2) the underidentification test with the Kleibergen–Paap rk LM statistic to indicate if there was direct effect of mobile phone penetration on HL; (3) the weak identification test to prove that the association between the IV and the HL was strong enough to meet the correlation condition and to avoid large estimation errors caused by the weak correlation; and (4) the falsification test to verify if mobile phone penetration indirectly affected the number of Hep B treatment via a pathway other than HL. We replaced the outcome variable (number of Hep B treatments) with an alternative outcome measure (total number of NA suppliers), which reflects the dynamics between the NA supply and the number of Hep B treatments to a certain extent. The number of NA suppliers is not affected by the HL but could be affected by the potential confounders that might be correlated with the mobile phone penetration. For example, the economic and technological developments, which might contribute to the popularization of mobile phones, and the development of new NAs for more efficient and convenient Hep B treatment. We performed the same 2SLS regression as done previously, and checked if HL could still affect the total number of NA suppliers. The nonstatistically significant result would reject the potential channel for the IV to indirectly affect Hep B treatment by the potential confounders while confirming the exclusion restriction assumption of the IV estimation [31-33].

### Sensitivity Analysis

The exogenous condition of the IV variable was examined with the “plausibly exogenous IV” analysis framework proposed by Conley et al [34], which checked the robustness of IV estimation by relaxing the strict exogeneity with model 5 (Supplementary Material S1 in Multimedia Appendix 1). Given the diverse socioeconomic landscapes of the 31 provinces, the regional disparities might affect Hep B treatment as well. To explore the potential modification effect of these provincial attributes, we conducted the subgroup analyses with model 4. We divided the 31 provinces into two groups according to the mean values of the newly reported Hep B incidence, total population, GDP per capita, THE per capita, proportionate health expenditures of public health institutions, and OOP% from 2013 to 2020 (Table S3 in Multimedia Appendix 1). To exclude the potential influence of the COVID-19 pandemic that started in 2020, we deleted the 2020 data. The same IV method was adopted to reanalyze the new set of data from 2013 to 2019.

## Results

**Figure 2.**
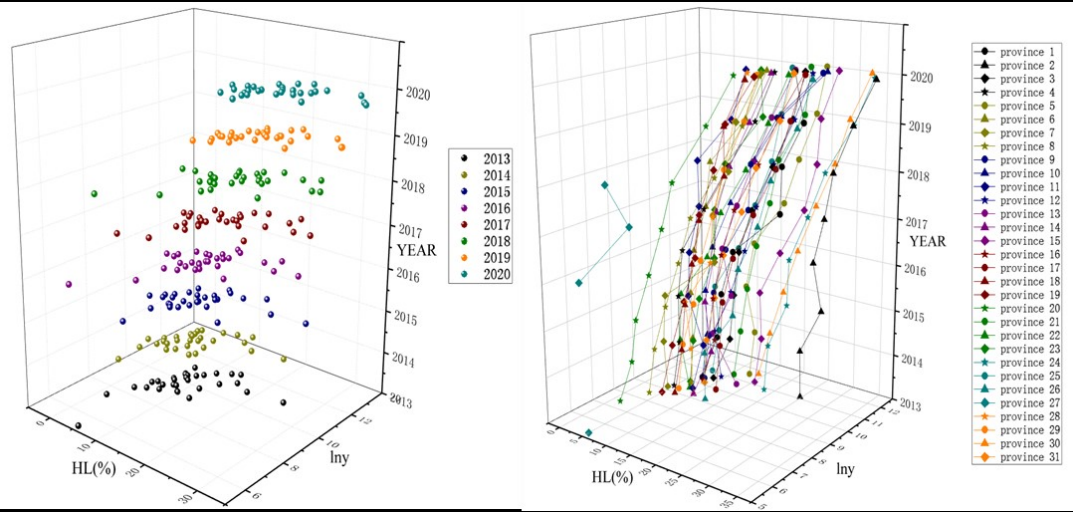
Scatter plots of HL(%) correlated with the number of the 12-month standard treatments of Hep B in 31 provinces from 2013 to 2020 in the cross-sectional dimension (A) and the time series dimension (B). HL(%): proportion of people who reached a health literacy score of >80; lny: logarithmic transformation of the number of standard 12-month antiviral treatments of Hep B.

In a specific year, the higher the HL, the higher the number of Hep B treatments in the cross-sectional dimension (Figure 2A). Each specific connection in the time series dimension represented the time-varying correlation between HL and the number of Hep B treatments in a specific province; the number of Hep B treatments increased with the improvement in HL (Figure 2B).

The results of the pooled OLS regression (model 1) showed that for a 1% increase in HL, the number of Hep B treatments increased by 7.68% (0.0768 = e^0^·^0740^ – 1; *P*<.001). After controlling the time-invariant inherent attributes of provinces, the results of province fixed effect regression (model 2) showed that for every 1% increase in HL in each province, the number of Hep B treatments increased by 4.55% (0.0455 = e^0^·^0445^ – 1; *P*<.001). After controlling the time-changing attributes, the results of the time fixed effect regression (model 3) showed that when HL increased by 1% in different provinces in each year, the number of Hep B treatments increased by 7.94% (0.0794 = e^0^·^0764^ – 1; *P*<.001). The IV estimation with the 2SLS regression (model 4) showed that for every 1% increase of HL in each province, the number of Hep B treatments increased by 7.15% (0.0715 = e^0^·^0691^ – 1; *P*<.001) (Table 1).

**Table 1.** The effect of HL (%)^a^ on the number of 12-month Hep B^b^ antiviral treatments.

	Pooled ordinary least square regression (Model 1)	Province fixed effect regression (Model 2)	Year fixed effect regression (Model 3)	IV^c^ 2SLS^d^ regression (Model 4)
HL (%)^e^ (SE)	0.074 (0.008)	0.045 (0.008)	0.076 (0.015)	0.069 (0.012)
Province fixed effect	No	Yes	No	Yes
Year fixed effect	No	No	Yes	No
GDP^f^ per capita	Yes	Yes	Yes	Yes
THE^g^ per capita	Yes	Yes	Yes	Yes
Health expenditures of public health institutions as a percentage of total health expenditure	Yes	Yes	Yes	Yes
OOP%^h^	Yes	Yes	Yes	Yes
Observations, n	244	244	244	244
*R* ^2^	0.82	0.74	0.83	0.72

a HL (%): proportion of people who reached a health literacy score>80.

b Hep B: hepatitis B.

c IV: instrumental variable.

d 2SLS: two state least square.

e All *P*<.001.

f GDP: gross domestic product.

g THE: total health expenditure.

h OOP%: proportionate out-of-pocket.

The least square dummy variable regression results showed that, compared with the reference province, many provinces demonstrated statistically significant differences, suggesting the necessity to adopt the province fixed effect model for estimation (Table S2 in Multimedia Appendix 1). The *F* test (*F*=0.5*, P*=.47) implied no time effect existed, so we performed the IV estimation with 2SLS regression based on the province fixed effect model (model 4).

The results of the endogeneity test (*χ*^2^=5.47, *P*=.02) showed that HL was an endogenous treatment variable, so IV estimation was appropriate. The results of the underidentification test (*χ*^2^=31.57, *P*<.001) showed that the IV satisfied the correlation condition. The weak identification test (Kleibergen–Paap rk Wald *F* statistic=63.71) was greater than 16.38 (critical value: 10%), indicating that the mobile phone penetration was not a weak IV. The falsification test showed no statistically significant effect of HL on the annual total number of NA suppliers (*P*=.51), which validated the assumption of restriction exclusion. Adopting model 5, the effect estimation interval of HL drawn from the Union of CIs method was (−0.0331, 0.0960), which included the estimation of model 4 (0.0715). This indicated that the deviation of IV estimation was within a tolerable range. The subgroup analysis found no statistically significant effect of HL on the number of Hep B treatments in the provinces with higher newly reported Hep B incidence and lower per capita GDP (Table S3 and Figure S2 in Multimedia Appendix 1). Data from 2020 when the COVID-19 pandemic emerged was excluded as they might affect both HL and Hep B treatments. Analysis of the 2013‐2019 data found that for a 1% increase in HL, the number of Hep B treatments increased by 5.19% (0.0519 = e^0.0506^ – 1, *P*<.001), which was slightly less than that of the original analysis result (7.15%).

From 2013 to 2020, the overall HL of the Chinese population increased from 9.5% to 23.2%, with an annual average increase of 2.0% [35-37]. By removing the 2020 data, the overall HL increased from 9.5% to 19.2% from 2013 to 2019; the annual average increase changed to 1.6%.

## Discussion

### Principal Findings

This study adopted different models to estimate the effect of improved overall HL of the population on the number of 12-month standard Hep B antiviral treatments based on a panel dataset of 31 provinces from 2013 to 2020. The regression results based on all models are consistent, suggesting a positive contribution of HL to the scale-up of Hep B treatment. The subgroups of provinces with higher newly reported Hep B incidence and lower GDP per capita were mainly those with lower levels of HL and Hep B treatment. The province with missing HL data was categorized in these two subgroups, thus reducing the statistical power, so that the positive impact of HL on Hep B treatment was not statistically significant. The pooled OLS regressions ignored the differences of the time-invariant inherent attributes among provinces and the potential changes in the macroenvironment over time. The marginal effect estimated based on model 2 is less than that based on model 1, indicating that ignoring the differences of the time-invariant inherent attributes among provinces might overestimate the effect. The marginal effect of the IV estimation is higher than the results of model 2, indicating underestimation due to the unobserved variables that might affect the number of Hep B treatments. Analysis of the 2013‐2019 data found a lower marginal effect, implying the overestimation brought by the COVID-19–related confounders in 2020.

### Comparison With Prior Work

Existing evidence shows that the number of 12-month standard Hep B antiviral treatments increased from 1,032,200 to 2,886,200 between 2013 and 2020 [38], with an annual average growth rate of 25.7%. During the same time, we found that the annual average increase of HL was 2%. Thus, for each 1% increase in HL, the number of treatments increased by 13%. In 2019, the number of 12-month standard Hep B antiviral treatments was 2,372,900 [38]; we found that the annual average increase rate of Hep B treatments was 21.6% during 2013‐2019 using the data generated from the existing study. Considering the 1.6% annual average increase of HL during the same time as shown in this study, we found that for each 1% increase of HL, the number of treatments increased by 13.5%. Both 13% for 2013‐2020 and 13.5% for 2013‐2019 were approximately double the IV estimations (ie, 7.15% and 5.19%, respectively). The gaps implied that if the endogeneity is ignored, the effect of HL on the number of Hep B treatments might be overestimated. This result might have been due to the unobserved factors that affected Hep B treatment other than HL, including the reduction of the treatment cost brought by the national pooled procurement of key NAs in 2019 [39]. The IV estimation based on the 2013‐2020 data was higher than that based on the 2013‐2019 data, indicating overestimation due to the confounding factor of the COVID-19 pandemic.

China has made great progress in reducing new infections of HBV in the past decades [40]. Moreover, the prices of NAs for Hep B antiviral treatment in China are the lowest in the world, which significantly promoted the scale-up of treatment. Hence, affordability is no longer a major barrier. However, there are still a substantial number of people infected with HBV to be identified, diagnosed, and treated. The low rate of treatment uptake is a crucial barrier for China to achieve the 2030 goal. Evidence showed that poor HL among a population prevents equal access to care, which is an underestimated public health problem globally [41-43].

### Policy Implications

Evidence generated in this study shed light on the next-step strategy to have a nationwide scale-up of Hep B treatment in China. Improving HL is a means to promote the scale-up of Hep B treatment. Improving the overall HL of the Chinese population, especially targeting the people with HBV with specific Hep B knowledge may be a feasible option to enable and facilitate the eligible individuals to ensure that they get diagnosed and adhere to the treatment. Improving HL has already been set as one of the national targets in the Healthy China 2030 blueprint [44]. The international society also recognizes HL as one of the key health promotion pillars for the achievement of the sustainable development goal targets, and the elimination of viral hepatitis as a major public health threat by 2030 is part of the Target 3.3 of the sustainable development goals [39].

To improve the overall HL of the Chinese population, it is possible to intervene from either the internal or the external aspects. From the internal aspect, interventions could target individual utility, the ability to access and utilize health information, and self-judgment [45]. These general education-based characteristics could be improved along with socioeconomic development [46]. From the external aspect, effective generation and dissemination of health information and specific Hep B knowledge could be a direct entry point. The main reason for the low treatment rate of Hep B in China is that many patients with surface antigen-positive HBV are not aware of their infection status [46]. Disseminate Hep B knowledge to these patients could help them to seek appropriate diagnosis and treatment.

In China, Hep B testing and treatment are financed through co-paid health insurance. Without a publicly funded universal screening, diagnosis, and treatment program, it is up to the individuals to approach rapid testing, and for the individuals with surface antigen-positive HBV to pursue diagnosis and treatment [45]. Raising awareness about Hep B through specific Hep B knowledge dissemination and helping patients with HBV infection to access appropriate health care services are critical for having eligible patients with infections diagnosed and adhering to the treatment. In the information era, efficient information generation and dissemination approaches such as mobile phones and new media are important external strategies for improving HL and raising awareness of Hep B [47,48]. For people with weak capacities in health information acquisition, comprehension, and utilization, it is necessary to consider interventions targeting both the internal and external factors that might influence their HL. Hence, it is necessary to ensure the accessibility and readability of health information and specific Hep B knowledge with targeted strategies to remove the barriers [49,50]. Strengthening the capacities of health information generation and dissemination with an efficient channel would result in a multiplier effect on promoting the scale-up of Hep B treatment through improving the overall HL of the population [51].

In addition to improving the HL of the public through raising awareness about Hep B, building an effective referral system and guiding the health care service utilization of HBV infections could be another approach. Currently, liver function tests and the HBV biomarker five-test have been included in the physical and premarital examinations of the healthy population, presurgery and preinvasive procedures tests, and obstetric/pregnancy monitoring. Even without a universal screening program for HBV, there is still a huge opportunity to increase the treatment rate in China if the above-identified infections could be appropriately managed. Most of these identified HBV infections were not followed up with, and the opportunity of keeping the patients with HBV infections eligible for treatment was lost. Even though there has been increased testing, with the absence of an appropriate referral system as well as assistance and guidance regarding different tiers of health care providers, the Hep B surface antigen-positive individuals screened using the above programs may not pursue diagnosis and treatment. An integrated management system for Hep B is critically needed to increase the treatment rate.

In some areas of China, a “four-in-one” model has been used for viral hepatitis management. Where the primary care institutions set up dedicated positions for viral hepatitis management, the full-time staff oversee managing the reported cases shared by the local Centers for Disease Prevention and Control. They follow up with the new cases via telephone within 10 days of reporting, mobilize and assist those patients who have not yet been given a diagnosis to visit their designated hospitals for nucleic acid tests and treatment, and provide continued telephone follow-up for subsequent treatment and progress [52]. Such a mechanism should be expanded across the country and could be developed as a solution for China to increase the treatment rate, and ultimately to have all the eligible patients treated.

### Limitations

There are several limitations of this study. First, only hospitals with more than 100 beds were included in the CHPA databases. Considering that the functions of the primary care facilities in Hep B prevention and control—as defined by the Basic Standards of Healthcare Facilities and the National Guidelines for the Management of Chronic Hep B at Primary Care—are in line with each other, and are also consistent with the findings from the interview of the primary care practitioners in Beijing, primary care facilities with less than 100 beds in China do not have the function and necessary capacity for Hep B treatment in China. Hence, Hep B antiviral treatment in China is mainly provided by secondary and tertiary hospitals [53], with the consumption of NAs at the primary care level being currently limited. Neglecting the consumption of antivirals at this level of care would not bring significant bias to this analysis. Second, the treatment of Hep B in real clinical settings may not follow the guidelines of a standardized treatment course. The number of 12-month (person-year) standard NA treatments of Hep B is under the ideal assumption that patients are receiving adequate therapy, while the real situation is much more complicated. Some physicians might not prescribe the treatment per the guidelines, and some patients might not comply with the treatment. Third, with the absence of patient-level data, the number of observations (31 provinces in 8 years) is limited. The findings of our provincial-level analysis should be carefully interpreted. The provincial-level data-based analytical findings may not be applicable in individual-level data settings. Fourth, our analysis was based on the data of the overall HL, not the specific HL about Hep B. However, among the 6 dimensions of the overall HL measurement as defined by the Chinese Health Literacy Scale, except for safety and first aid, which are not related to Hep B, the other 5 dimensions all directly or indirectly affect the treatment of Hep B. Therefore, overall HL measurement is generally acceptable for this analysis. Fifth, due to the unavailability of COVID-19 prevention and control information, as well as the epidemic data at the provincial level, we were not able to perform the analysis with more recent data after 2020. Future studies could explore relevant resources to include the analysis of the potential impact of the COVID-19 pandemic on HL and the number of Hep B treatments. Lastly, different provinces may have heterogeneous responses to interventions of HL improvement via mobile phone penetration, and IV estimation is a local average treatment effect estimation, which may include higher weights of the provinces that are more responsive [54,55]. The sensitivity analysis with the “plausibly exogenous IV” approach ensured that the robustness of our IV estimation was acceptable. In addition, we ignored the missing province-year observations based on the assumption that the dropout of specific provinces in specific years was not correlated with the error term, that is, data missing is random. A nonrandom sample might result in a sample selection problem and cause biased estimators.

### Conclusions

This study validated the positive effect of improved HL on the scale-up of Hep B antiviral treatment in China at the provincial level based on national panel data and IV estimation. Enhancing resource allocation to HL improvement in the following aspects would be a feasible and effective approach to increase the treatment rate of Hep B in China. One is the efficient generation and dissemination of Hep B–related knowledge to the public, helping them to understand their health status, build awareness of Hep B, and have a healthier lifestyle as well as appropriate medical behavior. The other is the facilitation of health care service utilization and referral guide for the people infected with HBV who need care, to enable the eligible individuals to be diagnosed and treated appropriately and to reduce loss of follow-up. Enhancing resource allocation to improve the overall HL of the population and Hep B–specific messages to people infected with HBV would be a feasible and effective approach to scale-up the treatment of Hep B in middle- and low-income countries with limited resources, and contribute to achieving the 2030 global goal for the elimination of viral hepatitis as a major public health threat.

## Supplementary material

10.2196/58391 Multimedia Appendix 1 Supplementary material.

## Abbreviations

CHPA: China Hospital Pharmacy Audit
GDP: gross domestic product
HBV: hepatitis B virus
Hep B: hepatitis B
HL: health literacy
IV: instrumental variable
LSDV: least square dummy variable
NA: nucleoside/nucleotide analog
OLS: ordinary least square
OOP%: proportionate out-of-pocket
THE: total health expenditure
