# Supplementary material for: Effect of Health Literacy on Antiviral Treatment of Hepatitis B: Instrumental Variable Analysis
Source: JMIR Public Health Surveill. 2024 Dec 16;10:e58391. doi: 10.2196/58391 (PMC11665480; doi:10.2196/58391)
Supplement: Multimedia Appendix 1 [file publichealth-v10-e58391-s001.doc]

**Title:** Effect of Health Literacy on Antiviral Treatment of Hepatitis B:

Instrumental Variable Analysis

**Table S1** Six NAs approved and recommended by the national treatment guideline for treatment of Hep B in China

| NAs | Market time of originator | Compound patent status | Dosage form | Strength & dosage/day | Indication | National health insurance covered indication | EML | RL |
| --- | --- | --- | --- | --- | --- | --- | --- | --- |
| entecavir, ETV | 2005.12 | API/  intermediate preparation only | Tab/Cap | 0.5mg | Adult and 2 -<18 child Hep B | No restriction | 2018.11 | 2009 |
| tenofovir disoproxil fumarate,  TDF | 2014.06 | Expired in 2017 | Ta/Cap | 300mg | Adult and ≥ 12 child  Hep B; HIV | No restriction | 2018.11 | 2017 |
| tenofovir alafenamide fumarate,  TAF | 2019.01 | Expired in 2021 | Tab | 25mg | Adult and ≥ 12 child  Hep B | Chronic Hep B | N | 2020 |
| telbivudine, LdT | 2007.04 | Expired in 2019 | Tab | 600mg | Adult Hep B | Active Hep B; interruption of mother-  infant transmission | N | 2009 |
| lamivudine, LAM | 1999.01 | Expired in 2006 | Tab/Cap | 300mg(HIV)  100mg(HBV) | Combined with antiretrovirals for adult and child HIV; adult  Hep B | Active Hep B; interruption of mother- infant transmission | N | 2000 |
| adefovir dipivoxil,  ADV | 2005.05 | Crystalline form protection only | Tab/Cap | 10mg | adult Hep B | No restriction | N | 2009 |

**Notes**: NA= nucleoside/nucleotide analogues; EML=essential medicines list; RL=reimbursement list; Hep B=hepatitis B. Except for ETV and TDF, hepatitis B is the only indication for all NAs. Human immunodeficiency virus (HIV) infection is another indication of ETV and TDF, but considering that China implements the national free treatment policy of HIV, antivirals for HIV treatment are purchased separately by the disease control program, thus data of ETV and TDF extracted from China Hospital Pharmacy Audit specifically reflect the consumption of ETV and TDF for Hep B antiviral treatment. Private sector provision for HIV treatment is rare.

**Figure S1.** The time series graphs of HL (%) and logarithmic transformation of the number of standard 12- month antiviral treatment of Hep B (lny) in 31 provinces of China (2013-2020)


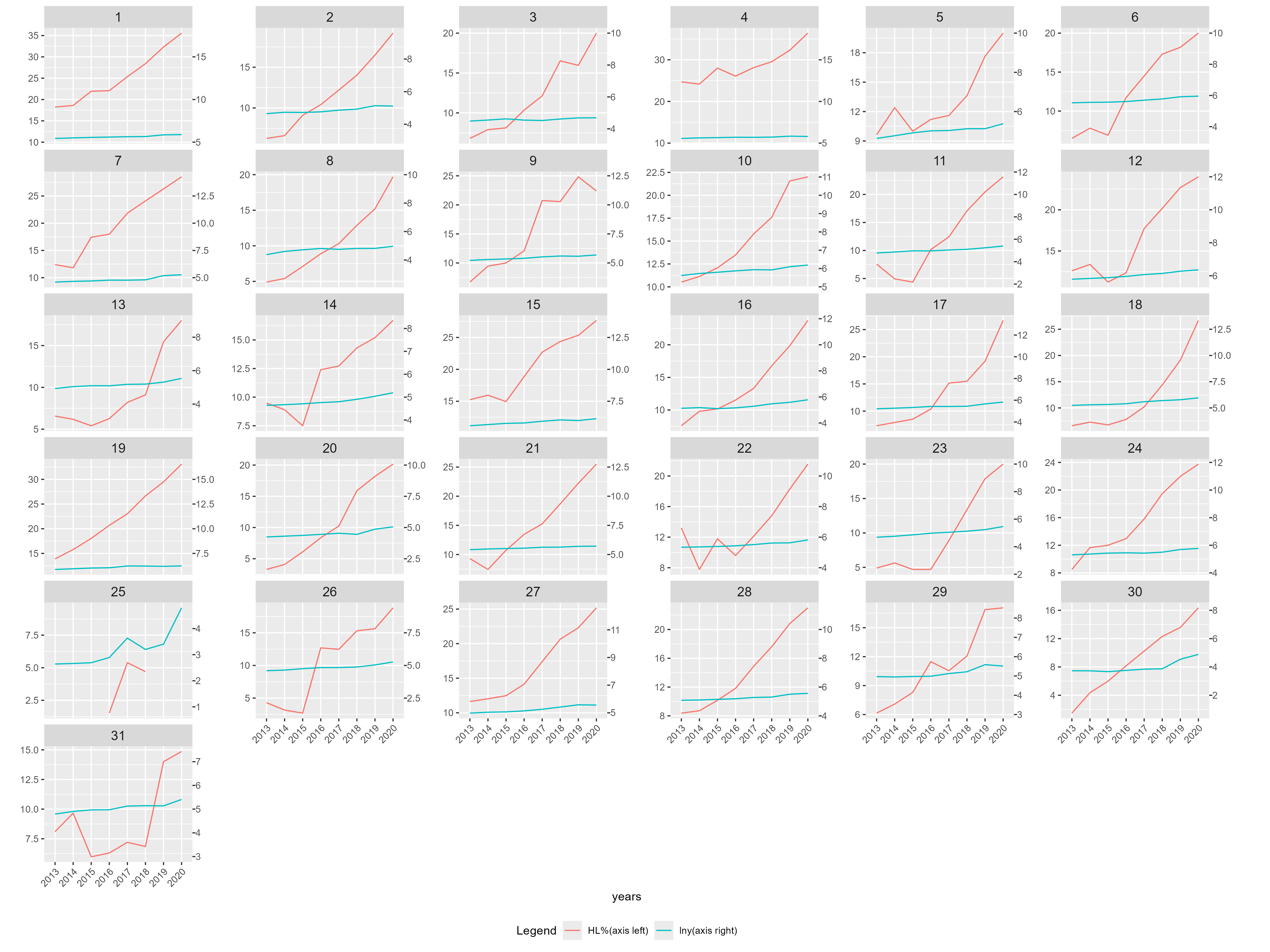


**Supplementary Material S1** Statistical analysis and sensitivity analysis methods

## IV selection

Internal factors of HL refer to the inherent attributes of individuals, such as gender, age, marital status, nationality, educational background, occupation (whether engaged in healthcare services), personal income, family income, chronic diseases, family size, lifestyle and healthcare philosophy etc. External factors of HL include the generation and dissemination of health information either within the health facilities or in the communities.

In recent years, social media and new health information technologies have become the mainstream dissemination approach for health information. As of 2020, the internet penetration was over 70% and there were over 1 billion internet users in China.[[1]](#footnote-2) The mobile phone has become the most common used channel for Chinese citizens to access health information. Compared with the traditional information dissemination channels, the mobile phone or iPad with multiple application programs may offer a faster, more convenient, plentiful and diversified health information dissemination approach, and facilitate tailor-made, efficient and convenient health education as well as patient follow-up management program.[[[2]](#footnote-3)](#bookmark1) Increased mobile phone penetration may enhance the HL by improving the efficiency of health information dissemination and patient management, and therefore facilitate Hep B treatment. Nonetheless, mobile phone penetration does not directly affect Hep B treatment.

## Models


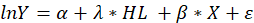
 （Model 1）

*Y* is the outcome variable and denotes the number of standard 12-month antiviral treatment of ep B. We performed a logarithmic transformation of *Y* to adjust for the skewed data. *HL* is the interpreted variable, *X* is the covariate, 𝛼 is the constant term and 𝜀 is the error term.


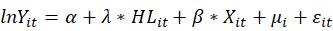
 （Model 2）


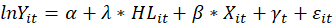
 （Model 3）

*i* represents provinces (numbered 1-31), *t* represents years (from 2013 to 2020), *Yit* represents the number of 12-month standard Hep B antiviral treatment of province *i* in year *t*. 𝜇𝑖 denotes province effect, independent of time. 𝛾*t* denotes the time effect, independent of provinces.


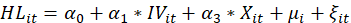
 （Model 4-1）


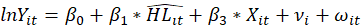
 （Model 4-2）


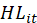
 is the HL of province *i* in year *t*,
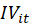
 is the mobile phone penetration,
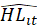
 is the fitting value obtained from the first stage regression,
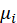
 and
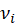
 are province effects;
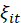
 and
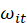
 are error terms.


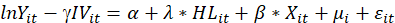
 （Model 5）

We obtained the prior information γ, which is the direct effect of IV on the outcome variable, reflecting the extent of deviations from the exact exclusion restriction. Strict exogeneity requires γ=0, but plausible exogeneity relaxes this restriction by defining γ to be near 0, but perhaps not exactly 0. θ is the sample estimated value of γ, which could be obtained from the sample data. This study followed the method used by *Liu C* et al., setting the range of γ as [0,2θ]. We adopted the Union of Confidence Intervals (UCI) to calculate the range of
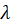
, which is the effect of HL on the number of treatment, under the plausible exogenous condition. We compared the range of 𝜆𝜆 with the estimations made by model 4 to judge the robustness of estimation. If the range of 𝜆𝜆 includes the estimation made by model 4, the deviation of IV estimation would be within a tolerable range.

**Table S2** LSDV regression results

|  | Coefficient | SE |
| --- | --- | --- |
| Residents’ HL scale | 0.0445^***^ | 0.00621 |
| Hep B morbidity | -0.000825 | 0.00123 |
| Population | 0.0000105 | 0.0000872 |
| GDP per capita | -0.00121^*^ | 0.000522 |
| THE per capita | 0.0206^**^ | 0.00735 |
| HEPH% | -0.0394 | 0.0224 |
| OOP% | -0.0122 | 0.0103 |
| 1.provs (reference) |  |  |
| 2.provs | -0.771^*^ | 0.312 |
| 3.provs | -1.184^***^ | 0.196 |
| 4.provs | -0.471^**^ | 0.167 |
| 5.provs | -0.670^*^ | 0.272 |
| 6.provs | 0.808 | 0.567 |
| 7.provs | -1.221^***^ | 0.212 |
| 8.provs | -1.090^***^ | 0.272 |
| 9.provs | 0.339 | 0.402 |
| 10.provs | 1.239 | 0.696 |
| 11.provs | -0.355 | 0.264 |
| 12.provs | 1.595 | 0.819 |
| 13.provs | 0.0588 | 0.329 |
| 14.provs | -0.994^***^ | 0.257 |
| 15.provs | 1.101^*^ | 0.534 |
| 16.provs | 0.251 | 0.307 |
| 17.provs | 0.340 | 0.516 |
| 18.provs | 0.667 | 0.679 |
| 19.provs | 1.312^***^ | 0.309 |
| 20.provs | -1.292^***^ | 0.241 |
| 21.provs | 0.701 | 0.388 |
| 22.provs | 0.622 | 0.451 |
| 23.provs | -0.451 | 0.261 |
| 24.provs | 0.772^**^ | 0.238 |
| 25.provs | -4.433^***^ | 0.479 |
| 26.provs | -0.728^**^ | 0.261 |
| 27.provs | -0.248 | 0.298 |
| 28.provs | 0.0582 | 0.172 |
| 29.provs | -0.145 | 0.259 |
| 30.provs | -2.611^***^ | 0.320 |
| 31.provs | -0.464 | 0.310 |
| _cons | 10.47^***^ | 0.378 |
| *N* | 244 |  |
| Adjusted *R*2 | 0.968 |  |

**Notes:** ^*^ *p* < 0.05, ^**^ *p* < 0.01, ^***^ *p* < 0.001; LSDV=least square dummy variable;

Hep B=hepatitis B; SE=standard error; HL=health literacy; GDP=gross domestic product;

THE =total health expenditure; HEPH%= health expenditure to public health institutions as a proportion of total health expenditure;

OOP%= proportionate out-of-pocket expenditure; *N*=number of observations

**Table S3** Subgroup analysis of the effect of the HL on the number of treatment of Hep B

| **Variables** | **Subgroups** | **No. of observations** | **Coefficient of HL** | **SE** |
| --- | --- | --- | --- | --- |
| Hep B morbidity | Low | 128 | 0.0817*** | 0.0127 |
|  | High | 116 | **0.0168** | 0.0188 |
| Population | Small | 124 | 0.0884*** | 0.0211 |
|  | Large | 120 | 0.0650*** | 0.0186 |
| GDP per capita | Low | 124 | **-0.0160** | 0.0484 |
|  | High | 120 | 0.0685*** | 0.0123 |
| THE per capita | Low | 128 | 0.0886** | 0.0307 |
|  | High | 116 | 0.0781*** | 0.0220 |
| Health expenditure to public health institutions as a proportion of total health expenditure | Low | 124 | 0.0739*** | 0.0187 |
|  | High | 120 | 0.0789* | 0.0323 |
| OOP% | Low | 124 | 0.0701*** | 0.0163 |
|  | High | 120 | 0.1139* | 0.0567 |

**Notes:** ^*^ *p* < 0.05, ^**^ *p* < 0.01, ^***^ *p* < 0.001; SE=standard error; HL=health literacy; Help B=hepatitis B; GDP=gross domestic product; THE = total health expenditure; OOP%= proportionate out-of-pocket expenditure; *N*=number of observations

**Figure S2**  The time series graphs of HL (%) and logarithmic transformation of the number of standard 12- month antiviral treatment of Hep B (lny) of subgroups


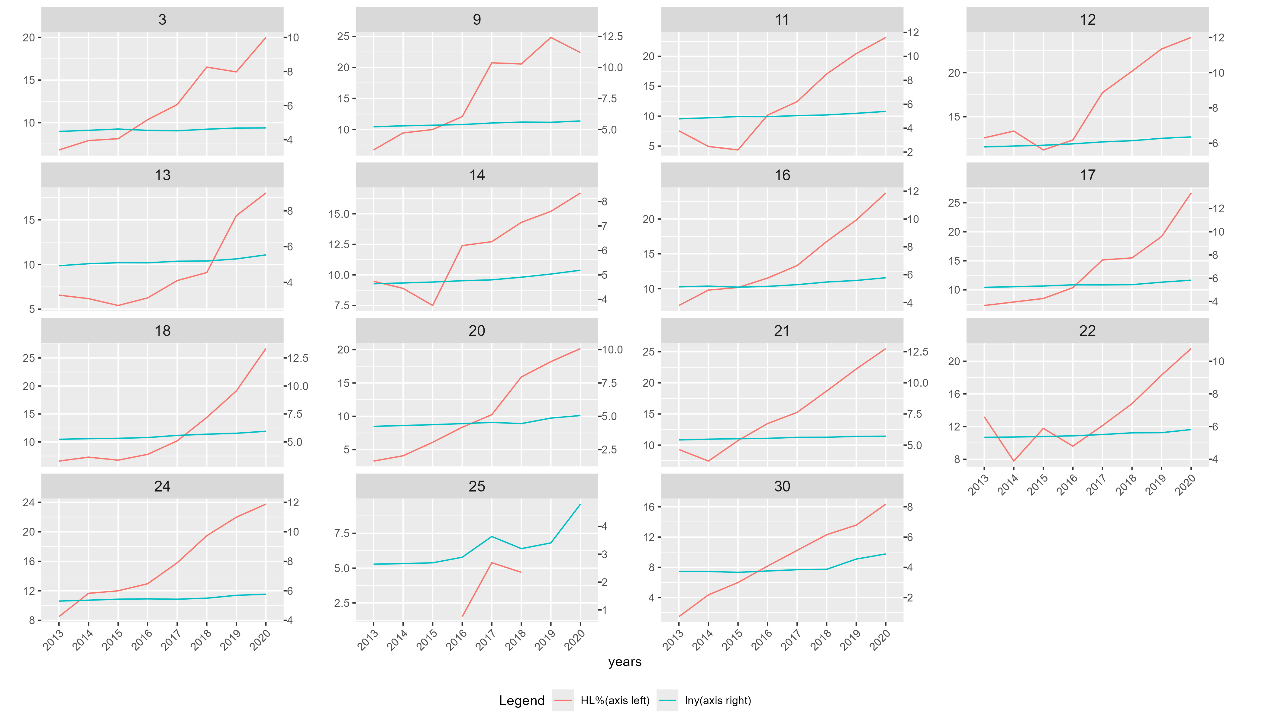

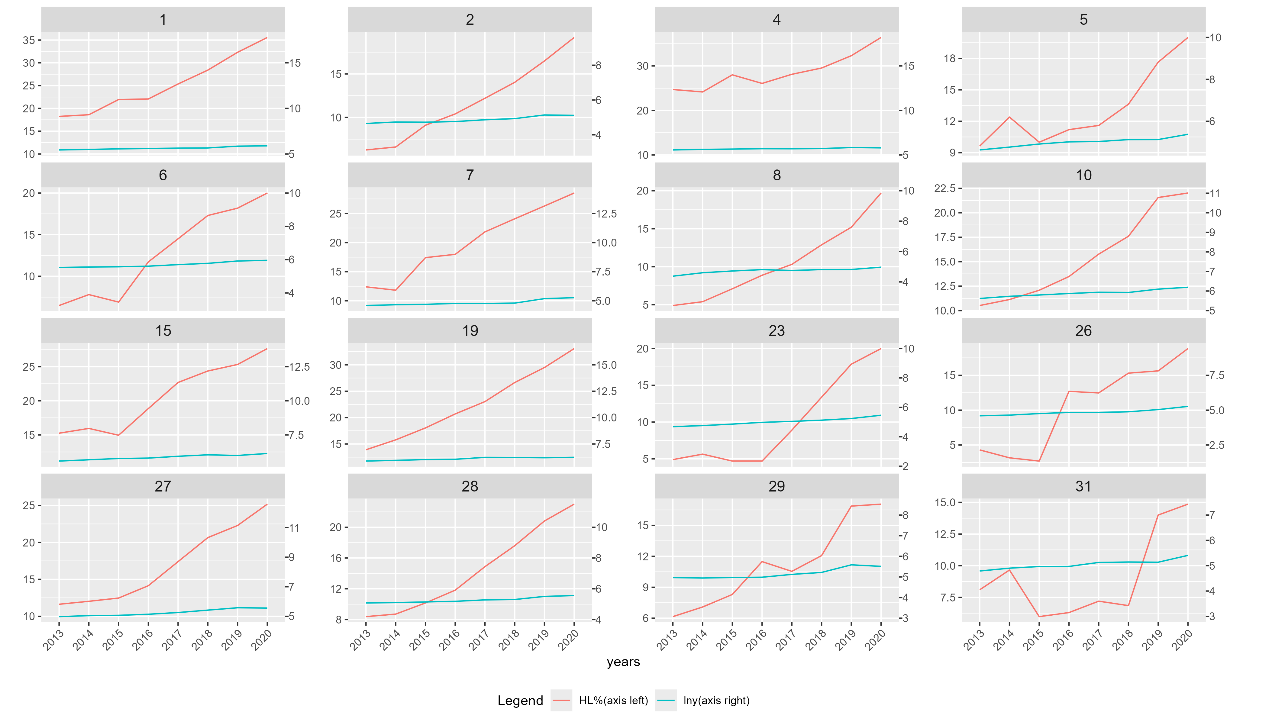


Provinces with higher level of Hep B morbidity Provinces with lower level of Hep B morbidity


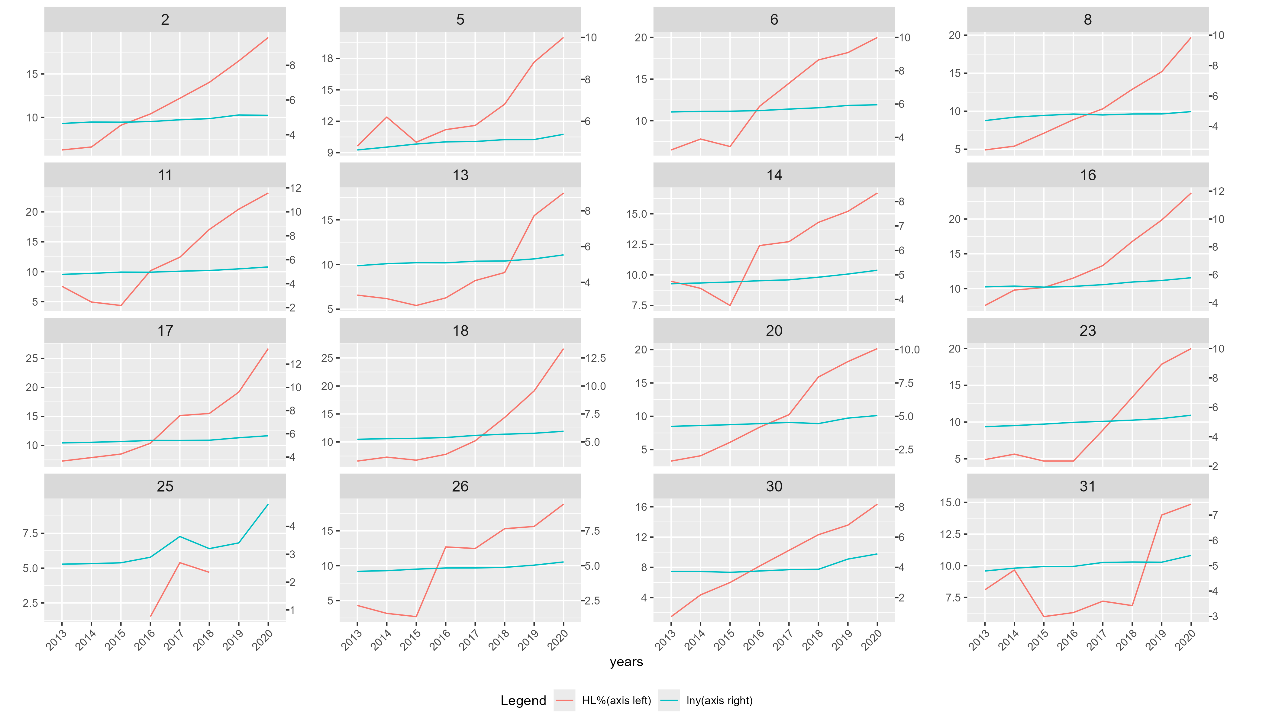

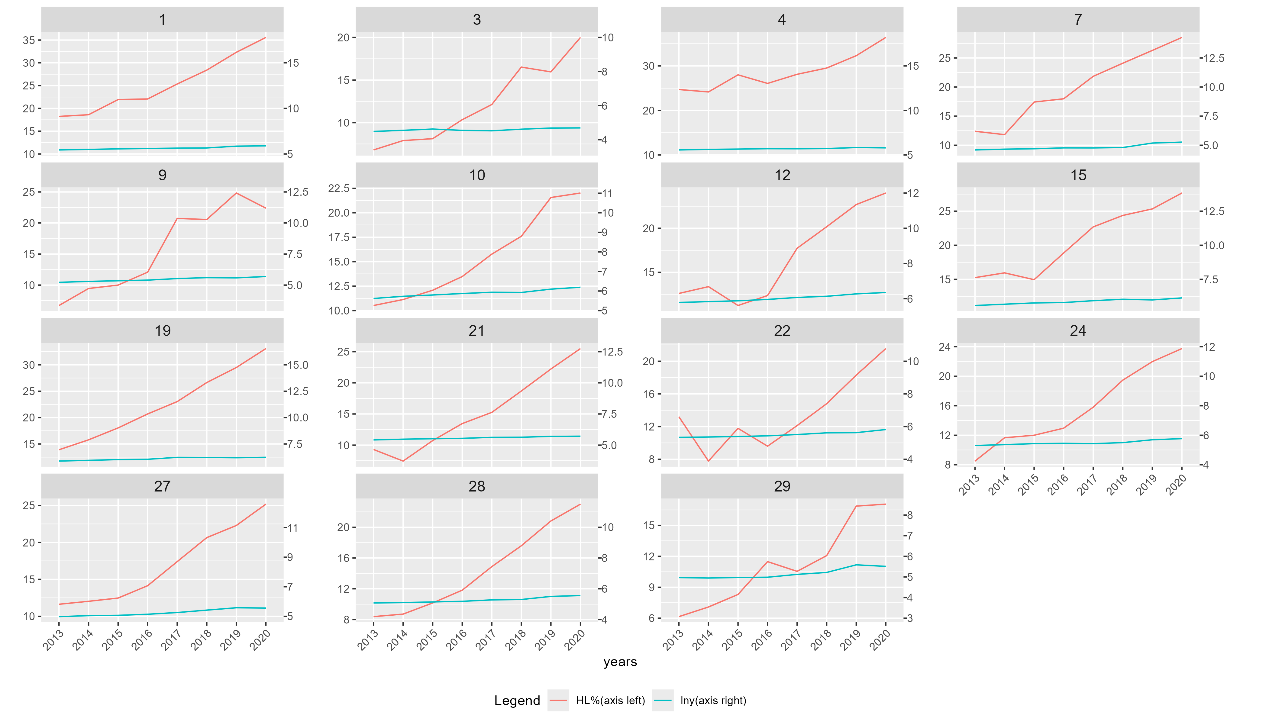
 Provinces with lower level of GDP per captita Provinces with higher level of GDP per capita

1. *National Bureau of Statistics*. [https://data.stats.gov.cn/.](https://data.stats.gov.cn/) Accessed January 6, 2023. [↑](#footnote-ref-2)
2. Mackert M, Mandell D, Donovan E, Walker L, Henson-García M, Bouchacourt L. Mobile Apps as Audience-Centered Health Communication Platforms. *JMIR Mhealth Uhealth.* 2021;9(8):e25425. [↑](#footnote-ref-3)
